# “I’ve been doing this for years”: the COVID-19 pandemic and family caregiver isolation and loneliness

**DOI:** 10.3389/fragi.2024.1376103

**Published:** 2024-05-31

**Authors:** Caitlin Sullivan, Judith B. Vick, Kasey Decosimo, Janet Grubber, Cynthia J. Coffman, Rebecca Bruening, Nina Sperber, Matthew Tucker, Joshua Dadolf, Nathan Boucher, Virginia Wang, Kelli D. Allen, S. Nicole Hastings, Courtney H. Van Houtven, Megan Shepherd-Banigan

**Affiliations:** ^1^ Center of Innovation to Accelerate Discovery and Practice Transformation (ADAPT), Durham Veterans Affairs Health Care System, Durham, NC, United States; ^2^ Department of Medicine, Duke University, Durham, NC, United States; ^3^ Veterans Affairs Boston Healthcare System, Cooperative Studies Program Coordinating Center, Boston, MA, United States; ^4^ Department of Biostatistics and Bioinformatics, Duke University Medical Center, Durham, NC, United States; ^5^ Department of Population Health Sciences, Duke University Medical Center, Durham, NC, United States; ^6^ Duke University, Sanford School of Public Policy, Durham, NC, United States; ^7^ The University of North Carolina at Chapel Hill, Chapel Hill, NC, United States; ^8^ Center for the Study of Aging and Human Development, Duke University, Durham, NC, United States; ^9^ Geriatrics Research, Education, and Clinical Center, Durham VA Health Care System, Durham, NC, United States; ^10^ Duke Margolis Center for Health Policy, Durham, NC, United States; ^11^ Durham VA Medical Center Mental Illness Research Education and Clinical Center, Durham, NC, United States

**Keywords:** caregivers, veterans, COVID-19 pandemic, loneliness, isolation

## Abstract

**Background:**

Family caregivers are family members or friends of care recipients who assist with activities of daily living, medication management, transportation, and help with finances among other activities. As a result of their caregiving, family caregivers are often considered a population at risk of experiencing increased stress, isolation, and loneliness. During the COVID-19 pandemic in the US, social isolation and decrease in social activities were a top concern among older adults and their family caregivers. Using secondary analysis of survey data as part of a multi-site implementation trial of a caregiver skills training program, we describe differences in caregiver experiences of loneliness before and during the COVID-19 pandemic.

**Methods:**

Health and wellbeing surveys of family caregivers were collected on 422 family caregivers of veterans before and during COVID-19. Logistic regression modeling examined whether the loneliness differed between caregiver groups pre vs during COVID-19, using the UCLA 3-item loneliness measure. Rapid directed qualitative content analysis of open-ended survey questions was used to explore the context of how survey responses were affected by the COVID-19 pandemic.

**Results:**

There were no significant differences in loneliness between caregivers pre vs during COVID-19. In open-ended responses regarding effects of COVID-19, caregivers described experiencing loneliness and social isolation; why they were unaffected by the pandemic; and how caregiving equipped them with coping strategies to manage negative pandemic-related effects.

**Conclusion:**

Loneliness did not differ significantly between pre vs during COVID-19 caregivers. Future research could assess what specific characteristics are associated with caregivers who have resiliency, and identify caregivers who are more susceptible to experiencing loneliness. Understanding caregiver loneliness could assist other healthcare systems in developing and implementing caregiver support interventions.

## 1 Introduction

In the U.S. there are more than 53 million family member or friend caregivers, and approximately 5.5 million are caregivers of veterans who provide assistance with veteran activities of daily living, medication management, transportation, and help with finances (Ramchand et al., 2014; AARP & National Alliance for Caregiving, 2020). Family caregivers are often considered a population at risk of experiencing increased stress, isolation, and loneliness as a result of their caregiving (Pearlin et al., 1990; Vasileiou et al., 2017; Kovaleva et al., 2018; Bramboeck et al., 2020). Social distancing, isolation, and stay-at-home orders in the first few months of the COVID-19 pandemic in the US comprised an unprecedented public health response (Luchetti et al., 2020; Talevi et al., 2020; Wang et al., 2020). While deemed necessary due to infectious risks of the SARs-CoV-2 virus, older adults and family caregivers reported that social isolation was a top concern during the pandemic (Chicago, 2021). Loneliness, defined as feeling as though the desire for relationships is not met due to infrequent social contact or inadequate amount of time with others, is one of the negative emotions that may have increased due to decreased social contact at the onset of COVID-19 (Heinrich and Gullone, 2006). Recent findings indicate that a large majority of caregivers experienced loneliness during the pandemic (Bristol et al., 2021); in fact, caregivers may be more vulnerable to loneliness than non-caregivers (Syed et al., 2021). Loneliness and social isolation have been associated with poor physical and psychological health outcomes exacerbating existing health conditions, and have been associated with premature death (Holt-Lunstad and Perissinotto, 2023).

Results from early research on family caregiver wellbeing during COVID-19 are inconsistent, but have mainly shown that caregivers experienced negative outcomes during the pandemic, including increased stress, anxiety, and concerns about finances, as well as a lack of outside help they normally receive (Beach et al., 2021; Carcavilla et al., 2021; Sheth et al., 2021). However, some other work shows that caregivers experienced no differences in mental or physical health (Ngamasana et al., 2023) or anxiety or depression from before or after the onset of COVID-19 (Seibert et al., 2022). Additionally, some caregivers who felt more hopeful during the pandemic had higher wellbeing (Onwumere et al., 2021). Many of these early studies are limited to comparing caregivers to non-caregivers or using cross-sectional quantitative data during the pandemic only, and were prone to recall bias with methods that relied on caregivers self-reporting perceived changes in wellbeing several months into the COVID-19 pandemic compared to the months before the pandemic (Miller et al., 2022).

As part of its healthcare system, the U.S. Department of Veterans Affairs (VA) administers one of the most comprehensive family caregiver support programs in the United States, offering caregiver group support and skills training, individual coaching, counseling, peer mentoring, and respite care. All of these services remained operational during the COVID-19 pandemic with pivots to virtual or phone-based delivery of supports and services. One study of family caregivers of veterans using a pre/post design and longitudinal data showed improved caregiver loneliness and wellbeing compared to before the COVID-19 pandemic; however, while improved, those domains were high before the pandemic and remained so during the pandemic (Miller et al., 2022). While these caregivers continued to experience negative pandemic-related wellbeing effects (Miller et al., 2022), it could be possible that the wellbeing improvements observed were due to VA caregiver services remaining active during the pandemic.

Supporting caregivers by decreasing social isolation and loneliness can impact their overall health and wellbeing as well as that of their care recipient (Edwards et al., 2020). Little is known of the context and experience of how the early phase of the COVID-19 pandemic “lockdown” (i.e., April—July 2020) impacted caregivers of veterans and how they managed negative pandemic-related effects, particularly in the areas of social isolation and loneliness. In this paper we describe differences for family caregivers of veterans in caregiver experiences of loneliness before and during the first few months of stay-at-home orders of COVID-19 pandemic. Understanding these initial impacts during temporary social disruptions could assist VA and non-VA healthcare systems in developing and implementing caregiver support interventions to alleviate pandemic-related effects.

## 2 Materials and methods

### 2.1 Study design overview

This study is a secondary analysis of survey data from a larger stepped-wedge cluster randomized trial evaluating the implementation of a VA caregivers skills training program, iHI-FIVES (implementation of Helping Invested Families Improve Veterans Experiences Study), part of the Optimizing Function and Independence VA Quality Enhancement Research Initiative (QUERI) (Van Houtven et al., 2014; Shepherd-Banigan et al., 2020). Using a pseudo-longitudinal design that has been used in other COVID-19 loneliness studies (Ernst et al., 2022), we examined cross-sectional surveys of different samples of family caregivers of veterans using the using the same loneliness measure for both samples.

### 2.2 Data collection

**FIGURE 1:**
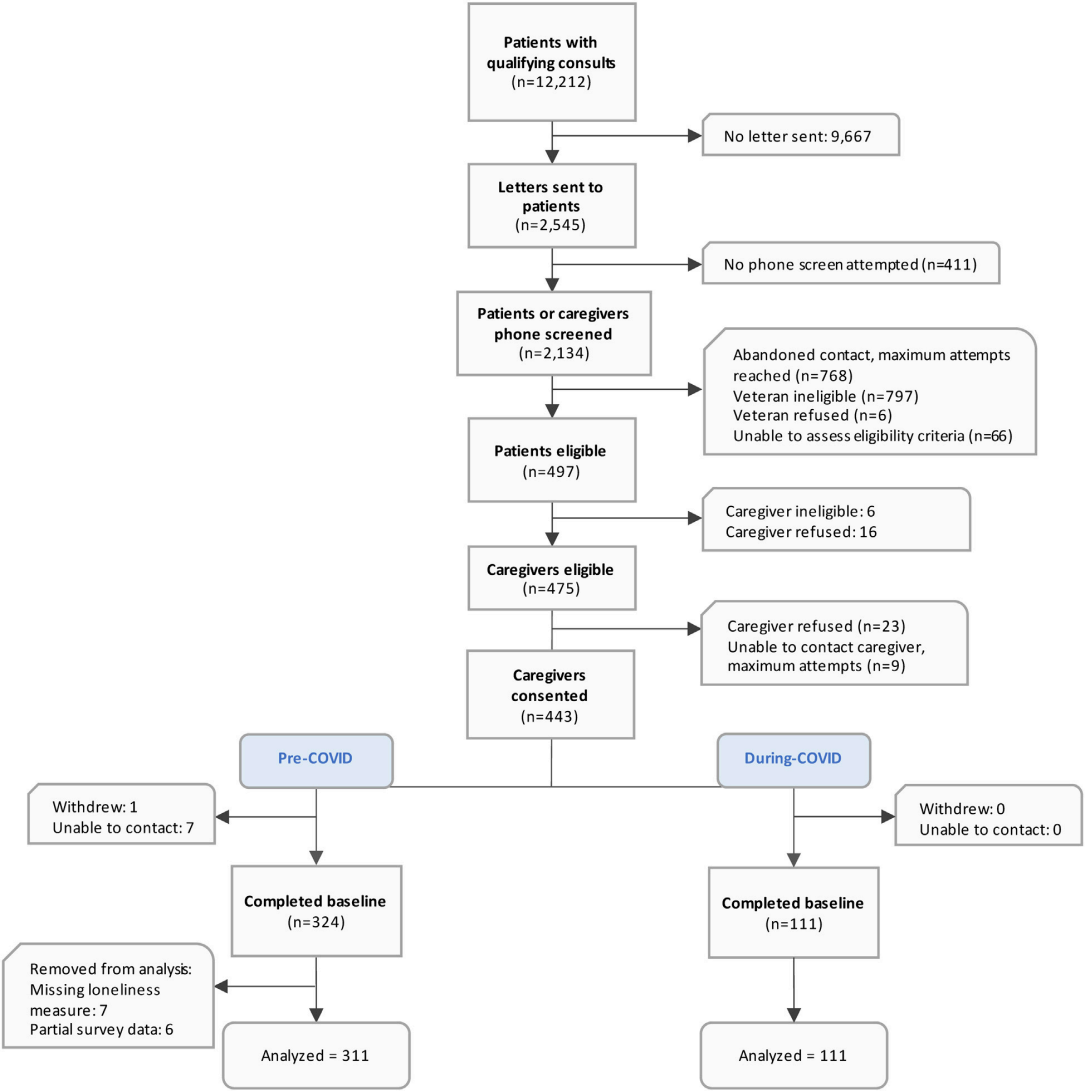
Study flow diagram.

Our study team collected survey data via telephone to assess the health and wellbeing of caregivers of veterans. Survey data was collected in “Wave 1” between 20 April 2018 to 17 January 2020 from caregivers who cared for veterans receiving healthcare at eight VA medical centers located throughout the United States, including the Pacific Northwest, the South, and Midwest/Northern regions (Figure 1– see notes) (Van Houtven et al., 2014; Ma et al., 2022). In March 2020, after a national emergency was declared for the COVID-19 pandemic, the study team rapidly developed open-ended questions for the survey to assess the effects of the pandemic on caregivers. We administered the same survey with the additional question to “Wave 2” caregivers meeting the same criteria as “Wave 1” from 21 April 2020 to 6 July 2020. Wave 1 (referred to as “pre-COVID-19”) and Wave 2 (referred to as “during COVID-19” during the first phase of the pandemic). Caregivers were not the same study participants; however, caregivers were sampled using the same data selection and were screened using the same methods.

### 2.3 Study participants

Caregivers of veterans were eligible to participate in the survey if the veteran received a referral to home and community-based services (homemaker home healthcare, home-based primary care, adult day healthcare, respite care, and veteran-directed care) at one of the eight participating VA medical centers within the study window (Ma et al., 2022). To identify eligible caregivers of veterans, the caregiver or veteran had to affirm that the telephone respondent was a family caregiver by endorsing that they “care for the veteran because of their ongoing health problems (for example, helping get around the house, bathing, or paying bills).” Excluded caregivers were those younger than 18 years, serving in a solely professional aide capacity, or reporting that their veteran was currently in a hospital, institution, or receiving hospice care). Eligible caregivers provided verbal informed consent. The project was approved by the IRB (Institutional Review Board) at the Durham VA healthcare system.

### 2.4 Measures

*Loneliness.* The outcome of interest was change in loneliness scores between the pre-COVID-19 and during-COVID-19 caregivers. To measure loneliness, we used the University of California, Los Angeles (UCLA) 3-item loneliness Likert scale measure (Russell et al., 1980; Hughes et al., 2004; Liu et al., 2020; Liu et al., 2020; Liu et al., 2020; Liu et al., 2020; Liu et al., 2020), which asks participants: “How often do you feel? a) lack of companionship; b) left out; and c) isolated?” Response options included never (coded as 0), rarely (1), sometimes (2), and always (3). We summed the ratings to create an overall score ranging from 0 to 9, with a higher score indicating a higher degree of loneliness. As in prior work, scores were dichotomized with scores of 4 or greater indicating experiencing loneliness (Rosenberg et al., 2021). Using this measure, we compared loneliness among two different groups of caregivers: 1) Wave 1 caregivers surveyed prior to COVID-19 from April 2018—January 2020 (pre-COVID-19)—included only quantitative survey data, and 2) Wave 2 caregivers surveyed during the COVID-19 pandemic from April 2020—July 2020 (during-COVID-19) which included the additional open-ended survey questions.

*Caregiver self-report of the effect of COVID-19 on their survey responses.* Since we anticipated the pandemic and stay-at-home orders might affect caregiver health and wellbeing, prior to collecting “Wave 2” of caregiver surveys, we added questions to the end of the existing survey: “We recognize that these are unique times to be asking you and other caregivers how you are doing. How much do you think the coronavirus (COVID-19) outbreak has affected your answers today?” As a follow-up question, caregivers were asked “tell me a little more about your answer, or why you chose that response.” The qualitative data was used to provide contextual information on how caregivers in the during-COVID-19 time period affected their survey responses. (Creswell et al., 2004; Fetters et al., 2013). The study team entered detailed verbatim notes of the participant responses into DatStat Illume, version 6.1 database. The lead author (CS) coded free text responses, with review from qualitative analysts (RB and NS) to develop consensus on the findings. Additional experts in health services research (MS, JV, CVH) reviewed the data, codes, and topics for rigor.

*Demographic data.* Explanatory variables of interest included demographic measures reported by the caregiver at the time of the survey: caregiver age, gender, race and ethnicity, marital status, education, perceived financial burden, work status, duration of caregiving, and relationship to the veteran. Most variables were dichotomized with the exception of race (Black, White, and multi-racial/other/unknown) and relationship to veteran (spouse/significant other, adult child, and other relative/friend).

### 2.5 Analysis

Descriptive statistics were calculated for sociodemographic characteristics of caregivers and the UCLA loneliness measure. Unadjusted and adjusted logistic regression models were fit to examine associations between self-reported loneliness between pre- and during-COVID-19 time periods. In addition to the indicator variable for COVID-19 time period, the adjusted logistic model included covariates of caregiver age, gender, race, relationship to the veteran, financial burden, and education. Descriptive analyses were conducted with SAS v.9.4 (SAS Institute, Inc, Cary, NC). Directed qualitative content analysis (DQICA) was used to analyze the open-ended survey questions of the ‘Wave 2’ during-COVID-19 caregivers. Using rapid qualitative analysis of open-ended data, we analyzed the verbatim notes thematically, focusing on caregiver feelings of isolation and loneliness to contextualize how much caregivers felt COVID-19 affected their survey responses (Hsieh and Shannon 2005). During this iterative process, we identified several emergent topics. As is common in rapid analysis, we did not develop a traditional codebook, but organized and summarized data within a matrix format (Hamilton and Finley, 2019). We reviewed all open-ended responses and narrowed down responses related to feelings of isolation and loneliness due to the COVID-19 pandemic. We individually read through the text and then through iterative discussion, we grouped text that addressed similar experiences. Then, within those topics, we further grouped the data to identify subtopics. Once we agreed on the primary and subtopics, we summarized the data and identified relevant quotes. The primary topics we identified were 1) caregivers had varied experiences related to loneliness during the COVID-19 pandemic (example subtopics: inability to receive outside support for Veteran; life is not any different), 2) strategies to cope with the pandemic (example subtopic: faith).

## 3 Results

### 3.1 Demographics

We analyzed 422 caregivers with 311 surveyed pre-COVID-19 and 111 surveyed during-COVID-19 during the initial phase of the pandemic (Figure 1). Most caregiver sociodemographic characteristics were similar across the pre- and during-COVID-19 groups (Table 1). Overall, the majority of participating caregivers were female, White, non-Hispanic; and more than half were 65 years or older. There were more adult children pre-COVID-19 caregivers compared to during-COVID-19 (31% compared to 22%) and more during-COVID-19 “other relative/friend” caregivers compared to pre-COVID-19 (24% compared to 11%). The majority of caregivers were not financially burdened; however, the proportion reporting financial burden decreased across the pre- (21%) and during-COVID (9%) survey respondents. Most caregivers completed more than a high school education, were not employed, and had been serving in their caregiving role for two or more years.

**TABLE 1:** Participant demographics.

	Pre-COVID-19 caregivers (Wave 1)		During-COVID-19 caregivers (Wave 2)		*p*-value^a^
	N = 311		N = 111		
Variable	n	(%)	n	(%)	
Age (y)^b^					0.80
<65	146	(47.71)	50	(46.30)	
≥65	160	(52.29)	58	(53.70)	
Gender					0.68
Female	279	(89.71)	98	(88.29)	
Race					0.97
White	217	(69.77)	78	(70.27)	
Black	56	(18.01)	21	(18.92)	
Multiracial/other/unknown^c^	38	(12.22)	12	(10.81)	
Ethnicity					0.82
Hispanic	18	(5.81)	7	(6.31)	
Relationship to Veteran					0.002
Spouse/significant other	181	(58.20)	60	(54.05)	
Adult/Child	95	(30.55)	24	(21.62)	
Other relative/friend	35	(11.25)	27	(24.32)	
Financial burden^b^ ^,^ ^d^					0.008
Yes	64	(20.85)	10	(9.35)	
Education					0.47
Completed high school education or less	76	(24.44)	31	(27.93)	
Work status now					0.52
Full/part time	76	(24.44)	25	(22.52)	
Length of caregiving					0.75
<2 years	69	(22.19)	23	(20.72)	
≥2 years	242	(77.81)	88	(79.28)	
Marital status					0.81
Married/living as married	227	(73)	80	(72.07)	
Self-rated health^b^					0.54
Good or better health	214	(69.03)	80	(72.07)	

^a^ Chi-squared test used to examine association of caregiver demographics with COVID, time period.

^b^ Missing: Age: Pre-COVID, 5; During-COVID, 3; Financial burden: Pre-COVID, 4; During-COVID, 4; Self-rated health: Pre-COVID, 1.

^c^ Includes the following or if more than White or Black was selected on check all that apply: American Indian or Alaska Native; Asian; Native Hawaiian or other Pacific Islander; Other (Specify); Don’t Know; Missing; Refused.

^d^ Financial burden: cutting back to pay bills or difficulty in paying bills.

Good or better health included self-rated health of responses of “Excellent”, “Very Good”, or “Good.”

*Loneliness* The mean loneliness score for pre-COVID-19 caregivers was 3.95 ± 2.62 compared to 3.41 ± 2.76 for during-COVID-19 caregivers with an estimated mean difference of 0.54 points (95% CI: −0.05, 1.14; *p* = 0.07). Among 311 pre-COVID-19 caregivers, 179 (58%) had a loneliness score of 4 or higher indicating loneliness (Rosenberg et al., 2021), compared to 52 of 111 (47%) during-COVID-19 caregivers. In the unadjusted analysis, there was no difference in odds of loneliness between pre- and during-COVID-19 caregivers (odds ratio 0.65; 95% CI: 0.42, 1.01; *p* = 0.052). Similarly, in the adjusted logistic regression model (Table 2) there was no difference in odds of loneliness between pre- and during-COVID-19 caregivers (OR = 0.80; 95% CI: 0.50, 1.29; *p* = 0.36). White, spousal, and higher financial burden caregivers were estimated to have higher odds of loneliness (see Table 2).

**TABLE 2:** Adjusted model odds ratios and associated 95% CI.

Logistic regression model results for examining association of loneliness in Veteran caregivers with COVID-19 time period			
	Adjusted model^a^		
Effect	Odds ratio	95% Wald	*p*-value
		Confidence interval	
COVID-19 time period			
During-COVID	0.80	(0.50, 1.29)	0.36
Pre-COVID	1.00	Reference	
Age			
<65	1.23	(0.76, 1.99)	0.40
65+	1.00	Reference	
Gender			
Male	0.76	(0.38, 1.53)	0.44
Female	1.00	Reference	
Race			
Black	0.46	(0.26, 0.80)	0.02
Multiple/other/unknown	0.84	(0.44, 1.59)	0.52
White	1.00	Reference	
Education			
Completed high school education or less	0.62	(0.39, 1.00)	0.05
Completed more than high school education	1.00	Reference	
Financial burden			
Not financially burdened	0.51	(0.29, 0.88)	0.02
Financially burdened	1.00	Reference	
Relationship to Veteran			
Adult child	0.56	(0.32, 0.97)	0.64
Other relative or friend	0.40	(0.21, 0.77)	0.05
Spouse	1.00	Reference	

^a^ 407 total included in adjusted model, 15 out of 422 had missing data.

*Caregiver self-report of the effect of COVID-19 on their survey responses.* Analysis of the open-ended responses about how much COVID-19 affected surveys includes only the “Wave 2” during-COVID-19 caregivers (n = 111). There were mixed experiences of negative, neutral, and positive effects of COVID-19, including feelings of isolation and loneliness as well as a focus on keeping a positive outlook. The qualitative responses were organized into two overarching topics: 1) caregivers had varied experiences related to loneliness during the COVID-19 pandemic and 2) coping strategies used during COVID-19.

1) Caregivers had varied experiences related to loneliness during the COVID-19 pandemic. Many caregivers reported feelings of isolation from remaining home on lockdown restrictions. Some caregivers dealt with a lack of social opportunities and loneliness from isolating from friends and family.

“*The virus is isolating people further than they already are as caregivers. I live with someone who is immunocompromised … you have to be cognizant of having family come visit, etc. I cannot hug or touch loved ones.” [CG1_Female_65 years and older]*

Some caregivers felt increased isolation and stress from taking on additional caregiving duties, and/or no longer had outside help to get a reprieve from their caregiving duties. Caregivers reported remaining isolated at home in fear of contracting the virus, and/or unable to leave the house since the veteran could not be left alone or could no longer attend day care.

“*I have no one to come in and help me with anything. Before, I had a caregiver taking him to dinner, so I had a break. I would have peace and quiet for an hour. Plus, I had to go to my support group and be gone a couple of hours once a week, had a care person for that, too. But they are not able to come.” [CG2_Female_65 years and older]*

On the other hand, while some caregivers felt increased isolation at home with their veteran, many caregivers described that they already lived in isolation and face similar restrictions prior to COVID-19 due to the veteran’s condition. Some caregivers were already following social distancing guidelines to keep the at-risk veteran safe prior to the pandemic.

“*The thing with this pandemic is you cannot get out and go all the time, and I cannot do that anyway with him [veteran care recipient]. I tell everybody: now y'all are living my life. I’ve been doing this for years.” [CG3_Female_65 years and older]*

More than half of the caregivers provided explanations as to why they were unaffected by COVID-19. Many were still living their lives and performing normal activities. Many were limited to their duties at home prior to COVID-19 and were used to feeling isolated, thus not impacted by stay-at-home restrictions. Some caregivers reported no change in their employment and/or were not facing additional financial difficulty from the pandemic. Some caregivers worked as essential personnel and continued to work while others lived in remote areas that had not yet been affected by the pandemic during the early pandemic (April - July 2020).

2) Coping strategies used during stay-at-home orders

“*I would have been staying home anyways, nothing very different than before.” [CG5_Female_Under 65 years]*

The final topic that emerged from the open-ended responses was the use of multiple coping strategies during COVID-19, including positive reframing and finding alternate ways to connect despite missing family and in-person interaction. During early phases pandemic, a few caregivers described how they relied on their faith or infused gratitude into their daily lives as a way to reframe their perspectives from negative to positive. For example, one caregiver discussed how they had to adapted to the new reality by reframing their perspective.

“*Frame of reference from before, my coping has changed but I can adjust. Life experience has taught me to adapt. I have high capability to adjust and adapt.” [CG7_Female_65 years and older]*

Other caregivers shared coping strategies such as engaging in artistic outlets and creatively connecting with family and friends. One caregiver described how she coped by identifying new ways to meet her needs for social interaction.

“*I try not to let it affect my mind or my happiness because there are always alternative ways of connecting … It’s about how creative you can be during this time.” [CG8_Female_Age not reported]*

Additional coping skills included relying on other relatives in the home, seeking additional help from the veteran where possible, relying on pets for interaction and stress relief, engaging in mental health counseling, and taking advantage of flexible or virtual work options. For example, one caregiver who worked outside of the home reported less stress balancing caregiving duties with the stay-at-home orders by being able to work remotely.

*“It’s a blessing in disguise. I can stay home (with work) and be closer to my husband.”* [*CG6_Female_Age not reported]*

## 4 Discussion

This study used a unique opportunity to assess loneliness among caregivers of veterans surveyed before and during the initial phase of the COVID-19 pandemic. Overall, there were no statistically significant associations between self-reported loneliness and pre- versus during-COVID-19 time period. The qualitative data showed there was a mixture of negative, neutral, and positive reactions during the pandemic which may explain the null quantitative findings. Research during the pandemic on older adults shows that not all groups were affected, and some subgroups experienced higher loneliness, thus demonstrating the importance of identifying at-risk groups that would benefit from targeted outreach (Kirkland et al., 2023).

While, on average, caregivers in our sample did not demonstrate high levels of loneliness compared with prior work (Shepherd-Banigan et al., 2020), some caregivers reported high levels of burden and isolation due to the pandemic. In general, caregivers were an already burdened population prior to March 2020, with many experiencing increased caregiving responsibilities during the pandemic (Savla et al., 2020). For example, some caregivers reported feeling isolated with the veteran because they were no longer were able to have visitors or paid workers in the home to provide respite care or help with daily tasks. For some caregivers, negative reactions to the sudden loss of paid help and additional resources highlighted the extent to which caregivers rely on respite and home healthcare.

Another possible explanation for the lack of difference in loneliness pre vs during the pandemic could be that some caregivers were already accustomed to feeling isolated at home caring for their loved one before the pandemic; therefore, a shift to life on lockdown did not change their circumstances (Hajek et al., 2021; Boucher et al., 2022). Since caregiving can be an isolating experience, many caregivers have developed coping strategies (Nicol et al., 2020). Caregivers have to be highly adaptable and flexible, and these skills may have benefited them during the pandemic. Caregivers with higher resilience experienced less burden during the pandemic (Palacio et al., 2020; Manzari et al., 2023). Additionally, prior research shows individuals experiencing loneliness who use positive coping mechanisms are able to reduce their feelings of loneliness (Fluharty et al., 2021). Building resilience and the use of positive coping strategies can mitigate negative psychological outcomes from caregiving, including loneliness.

Furthermore, since our sample included caregivers of veterans who received care at a VA Healthcare System, it is possible they remained connected to VA resources that may have helped them to offset loneliness and social isolation. As the VA is a healthcare system providing essential medical services, most resources and supports, such as the VA Caregiver Support Program, continued to operate throughout the pandemic. Also, some of the existing VA Caregiver Support Program services and supports utilized virtual modalities. A key takeaway from this work is to consider how caregiver support can be integrated into existing systems that have the resources and ability to provide continuous support to caregivers using various delivery modes.

### 4.1 Strengths

The current study exploited a unique opportunity to understand loneliness in similar groups of caregivers surveyed before and during the pandemic. Most prior research on family caregivers during COVID-19 reviewed psychological wellbeing of caregivers compared to non-caregivers and found that caregivers mainly experienced negative outcomes, such as increased anxiety, depression, and loneliness (Beach, Schulz, Donovan and Rosland, 2021; Liu et al., 2022; Liu et al., 2022; Liu et al., 2022). Limitations of prior research includes the predominantly cross-sectional nature of reviewing caregiver psychological outcomes solely during COVID-19 without a pre-COVID-19 baseline, as well as studies that compared caregivers to non-caregivers during COVID-19. Our study attempted to fill this gap by using a unique approach to quantitatively examine caregiver loneliness before and during COVID-19 and qualitatively by using open-ended responses to provide context from the quantitative results.

### 4.2 Limitations

While most of the demographics from caregivers surveyed pre and during COVID-19 are similar, we do not have repeated loneliness scores for the same caregivers to track over time. It is possible that the pre-COVID-19 caregivers were different from during-COVID-19 caregivers in unmeasured ways that we could not control for. The pre-COVID-19 caregivers had more financial burden and were more likely to be spouses and adult children (*versus* other relatives and friends) compared to during-COVID-19 caregivers. However, by and large the cohorts appear to be similar on most observed characteristics and were recruited using the same process. Overall, a limitation of pseudo-longitudinal studies is trajectories of individuals cannot be assessed over time so analysis is limited to the group-level; however, advantages to this design is that is less resource intensive than longitudinal studies while generating results comparable to traditional longitudinal designs (Bandyopadhyay and Mukherjee, 2021). Another limitation is the reduction in the number of caregivers from pre-COVID-19 to during-COVID-19, which could introduce sampling bias. However, in analysis we found minimal observed differences between groups. Additionally, our study surveyed caregivers of veterans and while the findings should be generalizable to non-veteran caregivers, it is possible there are differences in care recipient conditions and the level of caregiver support offered through VA (Ramchand et al., 2014). Finally, while our study provides contextual information from an open-ended question from caregivers surveyed after the pandemic began, it is not an in-depth qualitative analysis and limits our ability to further explore topics and subtopics.

## 5 Conclusion

This study identified diverse experiences of loneliness using quantitative and qualitative data and survey responses from similar groups of caregivers participating in a survey immediately prior to the pandemic (pre-COVID-19) and in the first months of the pandemic (during COVID-19). Our findings highlight the heterogeneity of family caregivers: some already felt isolated from being a caregiver and their social interactions did not change with lockdown at the start of the pandemic, while others more acutely felt the impacts of the lockdown, and many used coping strategies to manage. Future work identifying the key characteristics of caregivers less prone to loneliness in the face of mandated social isolation could have implications for developing preparedness plans for future public health events or natural disasters. Also, exploring how caregivers use coping strategies to offset loneliness, and developing tailored interventions can be an important tool to include in caregiver support programs. Finally, additional advocacy for use of services should be considered, and future research could review caregiver loneliness in the context of resource utilization and availability.

## Data Availability

The raw data supporting the conclusion of this article will be made available by the authors, without undue reservation.
